# Transcriptome-Wide Analysis of SAMe Superfamily to Novelty Phosphoethanolamine *N*-Methyltransferase Copy in *Lonicera japonica*

**DOI:** 10.3390/ijms16010521

**Published:** 2014-12-29

**Authors:** Yuan Yuan, Linjie Qi, Jun Yu, Xumin Wang, Luqi Huang

**Affiliations:** 1 State Key Laboratory of Dao-di Herbs, National Resource Center for Chinese Materia Medica, Academy of Chinese Medical Sciences, Beijing 100700, China; E-Mail: yuanyuan@icmm.ac.cn; 2 School of Biology and Pharmaceutical Engineering, Wuhan Polytechnic University, Wuhan 430023, China; E-Mail: qlj0810139@163.com; 3 CAS Key Laboratory of Genome Sciences and Information, Beijing 100029, China; E-Mail: junyu@big.ac.cn

**Keywords:** phosphoethanolamine *N*-methyltransferase, duplicated gene, *Lonicera japonica*, domestication, bud yield

## Abstract

The *S*-adenosyl-l-methionine-dependent methyltransferase superfamily plays important roles in plant development. The buds of *Lonicera japonica* are used as Chinese medical material and foods; chinese people began domesticating *L. japonica* thousands of years ago. Compared to the wild species, *L. japonica* var. *chinensis*, *L. japonica* gives a higher yield of buds, a fact closely related to positive selection over the long cultivation period of the species. Genome duplications, which are always detected in the domestic species, are the source of the multifaceted roles of the functional gene. In this paper, we investigated the evolution of the *SAMe* genes in *L. japonica* and *L. japonica* var. *chinensis* and further analyzed the roles of the duplicated genes among special groups. The SAMe protein sequences were subdivided into three clusters and several subgroups. The difference in transcriptional levels of the duplicated genes showed that seven *SAMe* genes could be related to the differences between the wild and the domesticated varieties. The sequence diversity of seven *SAMe* genes was also analyzed, and the results showed that different gene expression levels between the varieties could not be related to amino acid variation. The transcriptional level of duplicated PEAMT could be regulated through the SAM–SAH cycle.

## 1. Introduction

Protein methylation is catalyzed by *S*-adenosyl-l-methionine-dependent methyltransferases (SAMe); this posttranslational modification serves diverse cellular functions in plant growth and development [1], including flower scent and color development [2,3]. *SAMe* genes belong to a superfamily (SCOP53335) and include nearly 57 protein families of different domains [4]. Phosphoethanolamine *N*-methyltransferase (PEAMT) is the key enzyme of the plant Cho-synthesis pathway, which catalyzes all three of the methylations required to convert phosphoethanolamine to phosphocholine [5]. The upstream promoter sequence of *ZmPEAMT1* contained four kinds of putative cis-acting regulatory elements, including stress-responsive elements, phytohormone-responsive elements, pollen developmental special activation elements, and light-induced signal transduction elements, as well as several other structural features in common with the promoter of rice and *Arabidopsis* homologies [6]. The temperature-sensitive male sterility and salt hypersensitivity caused by PEAMT silencing in *Arabidopsis* [7]. Although studies on *SAMe* genes have provided great insight into the evolution of plant growth and development, our understanding of the mechanisms that control it is still poor.

The buds of *Lonicera japonica* are important in Chinese foods and medical materials; their active compounds include phenylpropanoids, terpenoids, and fatty acids. A new medicinal resource, *L. japonica* var. *chinensis*, is a variety with a greater content of active phenylpropanoid compounds but with lower bud yields. In transcriptome analysis, the minor differences between the two varieties are shown to be related to thousands of genes, including *SAMe* [8]. *SAMes* play important roles in the modification of such natural products as phenylpropanoids [9]. Caffeoyl-CoA *O*-methyltransferase (EC 2.1.1.104) is an enzyme involved in the biosynthesis of phenylpropanoids [10]. Six caffeoyl-CoA *O*-methyltransferases were cloned from *L. japonica* and its wild variety, and only three genes had greater transcriptional levels in the buds of *L. japonica*. The expressed profile showed that the transcript level of only one gene in buds of *L. japonica* was inferior to its ortholog from the *L. japonica* variety, and the contents of ferulic acid and quercitrin in *L. japonica* were also lower than that in its variety. Phylogenic analysis suggested that a functional divergence in paralogs may lead to variation in the gene function that controls the content of the active compound.

The *L. japonica* domestication process started thousands of years ago in China, and the content of one or more active compounds could not as the marker for plant breeding. Compared with the wild species, *L. japonica* var. *chinensis*, *L. japonica* had higher bud yields, a characteristic that is closely related to the positive selection inherent in the long span cultivation of *L. japonica* cultivation.

Genome duplications have always been detected in domestic species [11], and they have been responsible for the multifaceted roles of the functional gene [12]. Changes in gene duplicates expression are associated with differences in flowering behavior between wild and domesticated sunflowers [13] and, as well, other species. However, few studies have detailed the mechanisms through which duplications in a protein superfamily produce special characteristics in domesticated species.

In this study, we investigated the evolution of *SAMe* genes in 21 species, including *L. japonica* and *L. japonica* var. *chinensis*. We also further analyzed the roles that gene duplicates among special groups played during domestication, and we suggest that differences in the transcript levels of gene duplicates are related to variations in the amino acid or SAM–SAH cycle.

## 2. Results and Discussion

### 2.1. Global Phylogeny of SAMe Proteins

Using Superfamily, Interpro and BlastP, as well as information from public genome databases and our own transcriptome databases of *L. japonica*, we gathered 2354 non-redundant sequences that encode SAMe proteins from 21 different species (Table S1), representing a diverse taxonomic background. The results show that SAMe proteins are widely distributed among bacteria, fungi, animals, and plants. Among the 2354 sequences, 288 putative SAMe protein sequences are identified in *Selaginella moellendorffii*, compared to 6 SAMe proteins in *Escherichia coli* (Table 1).

We classified all SAMe protein sequences into three clusters (Figure S1). Some 48% of them are in cluster I. Those from the gymnospermae species are all found in cluster III; a few sequences from *Pinus taeda* and *Pseudotsuga menziesii* appear in cluster I. Pteridophyta, algae, monocotyledoneae, and dicotyledoneae species appear in all three clusters.

Cluster I is divided into 14 subgroups (I-1–14), with fully 52% of the sequences in subgroup I-1; only one copy from *Escherichia coli* appears in subgroup I-12. Almost all animals and fungi are found in subgroups I-1, 3, and 12, and one copy from *Culex quinquefasciatus* is in subgroup I-2. The sequences of algae, pteridophyta, dicotyledoneae and monocotyledoneae have a similar distribution among the subgroups of cluster I. Cluster II contains11 subgroups (II-1–11), and a few sequences from *L. japonica* are found in subgroup II-2.

**Table 1:** Copy number of *S*-adenosyl-l-methionine-dependent methyltransferases (*SAMe*) in 19 species.

Kingdom	Group	Class	Clusters * Species	Number of Copies																												
				I	1	2	3	4	5	6	7	8	9	10	11	12	13	14	II	1	2	3	4	5	6	7	8	9	10	11	III	Total
Animal			*Culex quinquefasciatus*	15	7	1	6	0	0	0	0	0	0	0	0	1	0	0	7	1	0	0	0	1	1	0	1	0	1	2	2	24
Bacteria			*Escherichia coli*	1	0	0	0	0	0	0	0	0	0	0	0	1	0	0	2	0	0	0	0	0	0	0	0	0	1	1	3	6
Fungus			*Penicillium marneffei*	8	4	0	3	0	0	0	0	0	0	0	0	1	0	0	4	0	0	0	0	1	1	0	1	0	1	0	2	14
			*Aspergillus nidulans*	10	4	0	5	0	0	0	0	0	0	0	0	1	0	0	4	0	0	0	0	1	1	0	1	0	1	0	2	16
Plant	Glymnospermae		*Pinus taeda*	2	0	0	0	0	0	0	0	0	2	0	0	0	0	0	0	0	0	0	0	0	0	0	0	0	0	0	2	4
			*Pinus pinaster*	0	0	0	0	0	0	0	0	0	0	0	0	0	0	0	0	0	0	0	0	0	0	0	0	0	0	0	1	1
			*Pseudotsuga menziesii*	1	1	0	0	0	0	0	0	0	0	0	0	0	0	0	0	0	0	0	0	0	0	0	0	0	0	0	5	6
	Algae		*Chlamydomonas reinhardtii*	34	16	6	4	5	0	0	0	0	0	0	4	2	0	0	16	0	0	0	0	3	3	0	2	0	5	3	13	63
	Pteridophyta		*Selaginella moellendorffii*	154	83	14	18	2	0	3	0	4	8	7	8	5	0	2	85	0	0	5	9	8	5	1	8	2	29	18	49	288
	Angiospermae	Dicotyle-doneae	*Glycine max*	145	85	11	7	1	0	3	0	13	11	1	5	4	0	4	135	0	0	14	12	16	15	2	11	5	29	31	53	225
			*Populus trichocarpa*	103	65	5	7	1	0	1	0	4	4	9	3	3	0	1	68	0	0	6	6	7	6	1	4	1	20	17	50	221
			*Arabidopsis thaliana*	66	36	5	6	1	0	2	0	2	2	6	2	3	0	1	40	0	0	4	3	5	3	1	1	0	11	12	13	119
			*Arabidopsis lyrata*	117	34	8	21	0	45	0	0	0	0	2	0	7	0	0	33	0	0	0	0	8	8	0	1	0	8	8	8	158
			*Vitis vinifera*	89	57	4	5	1	0	1	0	9	2	4	2	2	0	2	41	0	0	3	4	4	4	1	2	1	9	13	41	171
			*Lonicera japonica*	57	30	5	4	1	0	1	0	4	3	2	1	5	1	0	55	0	4	4	2	3	8	0	7	1	6	20	21	133
			*Lonicera japonica* var. *chinensis*	52	27	5	4	1	0	1	0	4	3	2	1	3	1	0	49	0	3	4	2	3	8	0	5	1	6	17	21	122
		Monocotyle-doneae	*Zea mays*	116	51	15	6	1	0	0	8	0	10	6	5	14	0	0	113	0	0	9	7	17	7	1	8	1	35	28	45	272
			*Sorghum bicolor*	68	36	6	6	1	0	0	5	0	2	4	2	5	0	1	48	0	0	2	3	6	6	1	2	1	14	13	44	160
			*Oryza sativa*	101	57	4	5	2	0	0	8	0	2	3	2	5	0	13	71	0	0	3	6	11	9	1	1	1	21	18	53	225
Total				1140	593	89	107	14	45	12	21	40	49	47	35	62	2	24	771	1	7	54	54	94	85	9	55	14	197	201	443	2354

* Clusters were showed in Figure S1.

### 2.2. Copying Genes of SAMe in L. japonica

Copying genes generate redundancy and create opportunity for evolutionary innovation [14]. From the Neighbor-joining trees, we compared the copy numbers of *SAMe* between sequences taken from *L. japonica* and *L. japonica* var. *chinensis* in the subgroups. Almost all the subgroups include the same number of *SAMe* copies of *L. japonica* and *L. japonica* var. *chinensis*, whereas the copy numbers for *L. japonica* are greater than those of *L. japonica* var. *chinensis* in the subgroups I-1, I-12, II-2, II-8, and II-11 (Table S2). The above results suggest that copying genes in these five subgroups plays an important role in *L. japonica*.

In subgroup I-1, 27 pairs of orthologs were found in *L. japonica* and *L. japonica* var. *chinensis*. Three redundant copies from *L. japonica* belong to a different protein family (PF05185, PF08498 and PF02353). In subgroups I-12, three pairs of orthologs were found and two redundant copies from *L. japonica* belonging to the same protein family (PF01209) (Figure S2). In subgroup II-2, all three pairs of orthologs belong to a protein family (PF03141) with one redundant copy from *L. japonica* (Figure S3). Four pairs of orthologs were found in subgroups II-8 (Figure S4), with three and one redundant copy from *L. japonica* and *L. japonica* var. *chinensis*, belong to a single protein family (PF03141). In subgroup II-11, seventeen pairs of orthologs were found, with three redundant copies from *L. japonica* belonging to one protein family (PF03141) (Table 2).

**Table 2:** Gene expression of floral developmental genes in *Lonicera japonica* and its wild variety.

Gene Name	Function in Floral Organ	AT NCBI Accession No.	FLJ		rFLJ	
			Accession No. *	RPKM	Accession No. *	RPKM
*FVE*	Vegetative to the flower-producing phases	AT2G19520	23402	0	563813	9.82
*FCA*		AT4G16280	172383	41.93	563294	57.57
*APETALA 1*	Morphogenesis	AT1G69120	146243	30.85	562895	0
*APETALA 3*		AT3G54340	124852	6.69	565304	0
*PISTILLATA*		AT5G20240	189255	6.34	568457	0
*AGAMOUS*		AT4G18960	191843	66.11	576158	57.57
*SEPALLATA*		AT1G24260	195015	8.09	571982	0
*SAHH*	Maintenance and recycling of *S*-adenosylmethionine dependent methylation	AT3G23810	183400	7.06	569411	0
*ADK*		AT3G09820	101959	323.33	388474	294.47

Abbreviations: AT, *Arabidopsis thaliana*; FLJ, *Lonicera japonica*; rFLJ, *Lonicera japonica*. var. *chinensis* (Watts.); RPKM, gene express RPKM; * identified sequences in database of *Lonicera japonica* and *Lonicera japonica*. var. *chinensis* (Watts.) in our group.

### 2.3. Expression of SAMe Genes in L. japonica Flowers

In order to further study the functional fate of the duplicated *SAMe* genes, we analyzed the transcript level of the *SAMe* genes based on *L. japonica* transcriptome data and real-time PCR. Besides II-2 subgroups, reads per kilo base per million (RPKM) of *SAMe* genes in subgroups with redundancy copies (Table S2) was greater in *L. japonica* than in *L. japonica* var. *chinensis*. In subgroup II-11, the total RPKM of the *SAMe* genes was 7.62-fold greater in *L. japonica* than that in *L. japonica* var. *chinensis*.

After duplication, both copies continue functioning when natural selection favors duplicated protein function or expression, or when mutations make them functionally distinct before one copy is silenced [15]. Approximately 50% of paralogs were differentially expressed and thus had undergone expression sub-functionalization by Soybean RNA-seq [16]. The RPKM of paralogs (FLJSAMT37 and FLJSAMT132 in I-1 subgroup) in the buds of *L. japonica* were 52.42 and 12.81, respectively. These differentially expressed copying genes in *L. japonica* could have undergone expression sub-functionalization or neo-functionalization.

We also analyzed the difference in gene expression between buds and flower1 of *L. japonica*. Buds had white or red petals that had not yet bloomed into a full-sized flower, and flower1 have white petals that had bloomed into a full-sized flower. Because of stable flower yields in buds and flower1, the differences in the transcriptional levels of genes in buds and flower 1 related to the flower yields should not be of any significance. Among the total eight *SAMe* genes, only seven *SAMe* genes, including FLJSAMT37, had different transcriptional levels between *L. japonica* and in *L. japonica* var. *chinensis*, but not between buds and flower 1 (Table 3), suggesting these genes could be related to the difference between the yields of flowers from the wild and from the domesticated varieties.

We further validated some above-mentioned *SAMe* genes as representatives using qRT-PCRs, and the results are consistent with the RNA-seq data (Table S3).

**Table 3:** Gene expression and amino acid variation of *SAMe* genes in subgroups.

Subgroups *	Orthologs Pfam	Gene	RPKM			Amino Acid Variation
			FLJ Bud	FLJ Flower1	rFLJ Bud	
II-2	PF03141	FLJSAMT59	120.99	106.17		365A/E
		rFLJSAM40			248.90	
II-8	PF03141	FLJSAMT53	495.65	860.77		275H/R, 289N/D, 441D/E, 623A/E
		rFLJSAMT30			0	
II-11	PF03141	FLJSAMT51	68.79	82.94		none
		rFLJSAMT28			0	
	PF03141	FLJSAMT73	151.06	153.34		none
		rFLJSAMT87			0	
	PF03141	FLJSAMT77	118.24	153.34		none
		rFLJSAMT97			0	
I-1	PF01135	FLJSAMT12	141.97	121.13		197V/I
		rFLJSAMT2			0	
	PF02005	FLJSAMT36	77.75	54.43		49E/Q, 299L/S, 599V/A
		rFLJSAMT45			0	
	PF02353	FLJSAMT37	52.42	60.38		none
		rFLJSAMT24			5.90	

Abbreviations: FLJ, *Lonicera japonica*; rFLJ, *Lonicera japonica*. var. *chinensis* (Watts.); FLJ bud, had white petals and had not yet bloomed into a full-size flower; FLJ flower1, had white petals and had bloomed into a full-size flower; rFLJ bud, had red petals and had not yet bloomed into a full-size flower; * Subgroups were showed in Figure S1; II-2, subgroups 2 in the second cluster; II-8, subgroups 8 in the second cluster; II-11, subgroups 11 in the second cluster; I-1, subgroups 1 in the fiest cluster.

### 2.4. Phosphoethanolamine N-Methyltransferase (PEAMT) in L. japonica Domestication

In plants, *SAMe* occurs as small superfamilies with defined roles for each of its members in flower development. *Arabidopsis* histone methyltransferase is crucial for both sporophyte and gametophyte development [17]. The transcript pattern showed that *Arabidopsis O*-methyltransferase is related to the developmental changes in flowers [9]. Acyltransferase was shown to be specifically expressed in anther tapetum cells in the early stages of flower development [18]. The *in vitro* substrate specificity and the *in vivo* RNAi-mediated suppression data of the corresponding gene suggest a role of this cation-dependent CCoAOMT-like protein in the stamen/pollen development of *A. thaliana* [19].

Because of a higher transcript level and greater number of copies in *L. japonica* than in *L. japonica* var. *chinensis*, a phosphoethanolamine *N*-methyltransferase (PEAMT, FLJSAMT37, Table S5) was selected to determine whether or not the duplicated gene in *L. japonica* domestication affects flower development. PEAMT has a central role in phosphatidylcholine biosynthesis via the methylation pathway [7]. Studies have shown that the synthesis of phosphatidylcholine is affected by the plant growth regulator indole-3-acetic acid [20], suggesting that phosphatidylcholine has a fundamental function in plant growth and development.

Phosphatidylcholine is also the immediate precursor of many phospholipids [21] and catalyzes the hydrolysis of phospholipids in the cell membrane into phosphatidic acid and polar free heads [22]. Increased expression of phospholipase D, which hydrolyzes membrane lipids to generate phosphatidic acid and associated lipid changes, promotes root growth, flowering, and stress avoidance [23]. A phospholipase A1 catalyzes the initial step of jasmonic acid biosynthesis, which synchronizes pollen maturation, anther dehiscence, and flower opening in *Arabidopsis* [24]. PEAMT could exert a role in cell division and inflorescence meristem. Inhibition of PEAMT biosynthesis led to necrotic lesions in leaves, multiple inflorescences, sterility in the flower, and early flowering in short day conditions [25]. However, an increase in endogenous phosphocholine content during plant development improves the root meristem size, cell division, and cell elongation in *Arabidopsis* [26]. Two phosphatidylinositol/phosphatidylcholine transfer protein genes are predominantly transcribed in the development of the male gametic cells and/or the fertilization process [27]. Thus, we suggest that duplicated PEAMT in *L. japonica* domestication could affect the inflorescence development and flower yield.

Flower development has two phases: (1) the steps from the vegetative to the flower-producing phases and (2) flower morphogenesis. We selected sequences of *FVE*, *FCA*, *APETALA*, *PISTILLATA*, *AGAMOUS*, and *SEPALLATA* from *Arabidopsis thaliana* and obtained sequences of their orthologs from *L. japonica* and *L. japonica* var. *chinensis* using BlastX, pfam, and interpro analysis. *FVE* and *FCA* follow a single-phase transition between the vegetative and the flower-producing phases [28]. *APETALA* and *PISTILLATA* control the formation of petals and stamens during *Arabidopsis* flower development [29]. *APETALA* and *AGAMOUS*-like act redundantly to control the identity of the floral meristem [30]. The *SEPALLATA* subfamily also plays a crucial role in the development of all types of floral organs [31]. The transcript level of *FVE* and *FCA* in buds of *L. japonica* was inferior to that of *L. japonica* var. *chinensis*, whereas those of *APETALA*, *PISTILLATA*, *AGAMOUS*, and *SEPALLATA* were greater, suggesting stronger floral meristem and morphogenesis in *L. japonica* than their wide variety (Table 2). This is consistent with the results of PEAMT expression.

### 2.5. Sequence Diversity of SAMe between L. japonica and Its Wild Variety

In order to investigate the reason for the differential expression of *SAMe* in *L. japonica* and its wild variety, the sequence diversity in SAMe proteins from subgroups I-1, I-12, II-2, II-8, and II-11 were analyzed. Consensus contigs developed from *L. japonica* served as the basis for alignment to detect single-nucleotide polymorphism (SNP). The readings of the individual sequences, realigned to the consensus contigs, enabled detection of 57 SNPs and 5 indels in the *SAMe* of *L. japonica* and *L. japonica* var. *chinensis*, based on a total uniquely aligned read number > 20 and a contingency test *p*-value <0.01. Only 13 residues of amino acids are changed in SAMe proteins of *L. japonica* and *L. japonica* var. *chinensis*, whereas novel PEAMT does not has either SNP or an indel; neither was a change in the residues in amino acids seen (Table 3).

### 2.6. SAM–SAH Cycle Regulates PEAMT Activity

Phosphocholine is synthesized by three successive *S*-adenosyl-Met (SAM)-dependent *N*-methylations of the phospho-base phosphoethanolamine [22]. This pattern is presumably due to the very active re-synthesis of SAM from ATP and Met made possible by recycling adenosine and homo-Cys derived from SAH [32]. Poulton and Butt [33] suggested that the ratio of SAM to SAH could regulate caffeic acid *O*-methyltransferase activity in the leaves of sugar beet (*Beta vulgaris*). The expression and activities of two enzymes, adenosine kinase (ADK) and *S*-adenosylhomocysteine hydrolase (SAHH), are both required for the maintenance and recycling of *S*-adenosylmethionine-dependent methylation in plants [34,35]. Subcellular localization of SAHH and ADK in the cytosol with the phospho-base *N*-methyltransferase activities in spinach [33]. More transcript levels of SAHH and ADK were also found in buds of *L. japonica* than were found in those of *L. japonica* var. *chinensis* (Table 3). SAHH and ADK were found to accumulate in a similar pattern and were also found at high levels in inflorescence meristems, likely to support their higher rates of cell division [36]. Greater amounts of PEAMT were observed in buds of *L. japonica* than in its wild variety, a finding consistent with the abundance of ADK and SAHH observed in these samples. These results indicate a positive correlation among transcript levels of PEAMT, ADK, and SAHH, reflecting their respective contributions to methyl metabolism.

## 3. Experimental Section

### 3.1. Plant Material

Buds and leaves of six each *L. japonica* and *L. japonica* var. *chinensis* plants were sampled in May 2012. These plants are 5 years old and situated in the field in Linyi planting garden, Yate Co, Shandong, China. Buds samples have similar morphology and have not yet bloomed into a full-size flower.

### 3.2. SAMe Classification

We searched the adenosyl-l-methionine-dependent methyltransferase sequences of 21 species (Table S1) using the superfamily [4] and InterPro databases. The species include one animal, one bacterium, two fungi, two algae, three gymnospermae, two pteridophyta, seven dicotyledoneae, and three monocotyledoneae. *L. japonica* database derived from five normalized libraries of transcriptome analysis. Flower samples (corollas or all petals) were randomly collected from five independent 3-year old FLJ and rFLJ in Doudian plantation (Beijing, China) to construct transcriptome libraries.

A total of 16,723 plant adenosyl-l-methionine-dependentmethyltransferases (*SAMe*) extracted from the NCBI non-redundant protein database, the SCOP SUPERFAMILY database [37] and the Unipro database [38]. We compared all searching sequences against the above plant SAMe sequence with an *e*-value cut-off below 1e^−15^ using BlastP [39] to determine the SAMe proteins from the best reciprocal hits. The resultant ESTs were dealt with Perl scripts to remove any repeated sequences.

### 3.3. SAMe Annotation

Domain and motif analyses were performed by InterPro [40] and Pfam [41]. The protein sequence similarities of SAMe were analyzed by DNAMAN. All the *L. japonica* and *L. japonica* var. *chinensis* SAMe sequences were submitted to COG [42] to cluster the SAMe orthologous groups with a *p*-value cut-off below 10^−5^.

### 3.4. SAMe Phylogeny

We used the SAMe sequences to construct neighbor-joining trees using Mega 5.0 [43] and ClustalW2 [44], respectively, with a bootstrap value of 1000 replicates. Furthermore, we reconciled preliminary trees by setting the bootstrap value greater than 50% to yield a consensus tree even more credible.

### 3.5. Orthologs and Paralogs

To identify orthologs, we performed an all-against-all sequence comparison using BLAST with an *e*-value cut-off below 1e^−20^. The orthologs were then determined based on the best reciprocal hits [45]. We implemented a more stringent criterion: the alignment length percentage against the longer protein had to be above 80%.

### 3.6. Gene Expression Analyses and Experimental Validation

The gene expression profiling of *L. japonica* flowers was performed in a previous study [8]. The expression level was normalized with total mapped reads and the contig length, similar to RPKM method [46]. The RPKM value for each transcript was calculated as the number of reads per kilobase of the transcript sequence per million mapped reads [47].

Individual RNA samples extracted from the buds of six each *L. japonica* and *L. Japonica* var. *chinensis* plants were used to produce cDNAs for qRT-PCR, including reactions without reverse transcriptase. The PrimerScript 1st Strand cDNA Synthesis Kit from Takara (Tokyo, Japan) was used, according to the manufacturer’s instructions. Gene-specific primers were designed using Primer 3 [48]. The primers are shown in Table S4. The amplifications were carried out with a 1 min incubation at 95 °C followed by 35 cycles at 95 °C for 15 s, 57–60 °C for 30 s and 68 °C for 30 s. The lengths of PCR products ranged from 100 to 250 bp. FLJ18S was chosen as an endogenous control in studying gene expressions in various bud samples of *L. japonica* and *L. Japonica* var. *chinensis*. The specificity of amplification was assessed by melting curve analysis, and the relative abundance of genes was determined using the comparative Ct method as suggested in ABI 7500 Software v2.0.1 (ABI, California, CA, USA).

### 3.7. SNP Identification, Validation and Sequences Diversity

Reads of *L. japonica* var. *chinensis* in the transcriptomes were mapped to FLJSAMes nucleotide sequences by BWA [49] and homozygous FLJSAMes SNPs were prepared by SAMtools [50]. Homozygous rFLJSAMes SNPs were also prepared by mapping the *L. japonica* reads to the FLJSAMes nucleotide sequences. Nucleotide sequences of FLJSAMes and rFLJSAMes were BLAST each other to find the best hits and matching position show the same variants count to candidate SNPs. SNPs with genotypic variants of the position within more than two reads or frank SNPs within 60 bp were removed. The filters we used to find the variants we could consider true SNPs are as follows: The minimum coverage of the position was eight reads and the minimum average quality of the bases was 20.

## 4. Conclusions

All SAMe protein sequences were classified into three clusters and several subgroups. Almost all subgroups have the same number of *SAMe* gene copies of *L. japonica* and *L. japonica* var. *chinensis*, whereas copy numbers of *L. japonica* are higher than *L. japonica* var. *chinensis* in the subgroups I-1, I-12, II-2, II-8, and II-11. The difference in transcriptional levels of the duplicated genes showed that seven *SAMe* genes could be related to the differences between the wild and the domesticated varieties. The sequence diversity of seven *SAMe* genes showed that the different expressed levels between varieties could be related to variations in the amino acid sequences. However, in the case of those containing PEAMT that had neither SNP/indel nor changes of amino acid residues, the transcript levels of PEAMT could be related to ADK and SAHH, reflecting their respective contributions to methyl metabolism.

## Supplementary Materials

Supplementary materials can be found at http://www.mdpi.com/1422-0067/16/01/0521/s1.
